# Giant pulmonary hydatid cyst causing a mediastinal shift in a child

**DOI:** 10.1590/0037-8682-0599-2022

**Published:** 2023-02-20

**Authors:** Yener Aydin, Ali Bilal Ulas, Atilla Eroglu

**Affiliations:** 1 Ataturk University, Medical Faculty, Department of Thoracic Surgery, Erzurum, Turkey.

**FIGURE 1:**
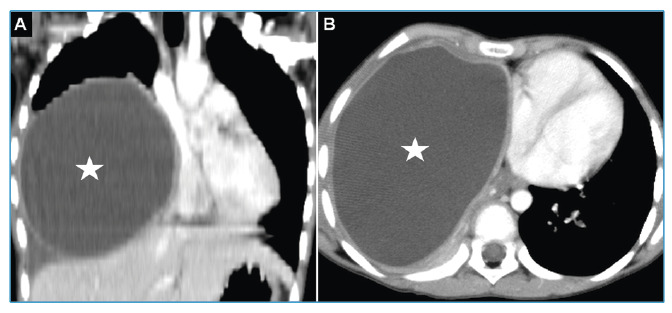
Contrast-enhanced thoracic CT coronal **(A)** and axial sections **(B)** shows a giant hydatic cyst of approximately 9 cm x 12 cm x 13 cm in the lower lobe of the right lung (arrows) and a shift to the left in the mediastinum.

A 9-year-old girl presented with a cough and dyspnea. Thoracic computed tomography (CT) detected a giant cystic lesion and mediastinal shift (Figure 1). The patient underwent cystotomy and capitonnage through a right thoracotomy.

Hydatid cyst disease continues to be an important public problem today. The causative agent of this disease is a parasite belonging to the cestode class, *Echinococcus granulosus* ^1^. Carnivorous animals are the parasite's definitive hosts, and the disease is transmitted via the fecal-oral route. The eggs of the tapeworm in the main host are excreted in feces. Water and food contaminated with these feces are taken up by intermediate hosts. The life cycle continues when carnivorous animals consume the cyst-containing internal organs of herbivorous intermediate hosts. Hydatid cysts grow faster in children than adults due to the elasticity of the lung^2^ ^,^ ^3^. Hydatid cysts may rarely cause a mediastinal shift. Children with pulmonary hydatid cysts should be treated surgically as soon as possible.
